# Uptake of Polyelectrolyte Functionalized Upconversion Nanoparticles by Tau-Aggregated Neuron Cells

**DOI:** 10.3390/pharmaceutics13010102

**Published:** 2021-01-14

**Authors:** Yo Han Song, Ranjit De, Kang Taek Lee

**Affiliations:** 1 Department of Chemistry, Gwangju Institute of Science and Technology (GIST), Gwangju 61005, Korea; yhsong@gist.ac.kr (Y.H.S.); deranjit@postech.ac.kr (R.D.); 2 Department of Life Sciences, Pohang University of Science and Technology (POSTECH), Cheongam-Ro 77, Pohang, Gyeongbuk 37673, Korea

**Keywords:** polyelectrolytes, upconversion nanoparticles, surface functionalization, tau aggregation, drug delivery, bioimaging

## Abstract

Tauopathy is the aggregation phenomenon of tau proteins and associated with neurodegenerative diseases. It metastasizes via the transfer of tau aggregates to adjacent neuron cells; however, the mechanism has not yet been fully understood. Moreover, if the materials used for designing drug delivery system to treat such neurodegenerative diseases do not undergo biodegradation or exocytosis but remains in cells or tissues, they raise concerns about their possible negative impacts. In this study, the uptake and delivery mechanisms of nano-sized carriers in tau aggregated neuron cells were investigated employing polyelectrolyte-functionalized upconversion nanoparticles (UCNPs) of diameter ~100 nm. Investigation through bioimaging was carried out by irradiating the particles with near-infrared light. Here, forskolin and okadaic acid were employed to induce tau aggregation into healthy neuron cells. It was noticed that the tau-aggregated neuron cells, when treated with relatively large sized UCNPs, showed uptake efficiency similar to that of normal neuron cells however their intracellular transport and exocytosis were impacted, and most of the carriers remained accumulated around lysosome. This demonstrates that metastasis mechanisms of tauopathy can get influenced by the size of carriers and are to be considered during their pharmacokinetic studies which is often not addressed in many drug delivery studies.

## 1. Introduction

In addition to Alzheimer’s disease (AD), aggregation of tau proteins is also reported in the brains of patients with frontotemporal lobar degeneration (FTLD), Pick’s disease, progressive supranuclear palsy, corticobasal degeneration, etc. and these neurodegenerative diseases are known as tauopathy [1,2]. Tau is a microtubule-associated protein, whose main function is to stabilize microtubules in axon of neuron cell, cargo trafficking and axonal growth [1]. Since this protein plays a pivotal role in regulating microtubule (MT) dynamics, e.g., controlling MT coupling, dynamic motion, spatial composition, etc., it is precisely controlled by various factors including post-translational transformations [3]. Therefore, when tau aggregation phenomena occur due to tau mutations (especially hyperphosphorylation) in neurons, serious neurodegenerative diseases are resulted [4].

Phosphorylation of tau in AD effected brain is 3 to 4 times higher than normal levels [5,6]. The degree of phosphorylation of tau is controlled by the impacts of proteins kinase and phosphatase (PP). Up-regulation of protein kinase or down-regulation of protein phosphatase is recognized as major factors in causing tau hyperphosphorylation. Among various protein kinases, protein kinase A (PKA) is one of the prominent kinases associated with tau phosphorylation [7,8]. Pre-phosphorylation of tau by PKA significantly facilitates the subsequent tau phosphorylation at multiple sites by serine/threonine protein kinase glycogen synthase kinase 3 (GSK-3) [9]. In relation to this tau hyperphosphorylation, the most potent inhibition of microtubule assembly in vitro is phosphorylation by PKA and GSK-3 [10]. Additionally, in post-mortem specimens of AD brain, total phosphatase activity was found to be reduced by half while protein phosphatase-2A (PP2A) and PP1 activities were reduced by 50% and 20%, respectively. It is reported that this phosphatase can play an important role in AD pathogenesis [11]. Among the various phosphatases involved in rescuing the biological function of tau by dephosphorylating hyperphosphorylated tau, PP2A plays pivotal role [12]. It is well known that inhibition of PP2A or upregulation of PP2A inhibitors by okadaic acid (OA) can stimulate tau hyperphosphorylation in cell lines and mice brains [13,14,15].

It has been reported that the movement of organelles, such as mitochondria, in axons can be impaired by tau pathology resulting neurodegeneration [16]. Neurotoxicity due to oxidative stress and free radicals, caused by the disruption of mitochondrial function, have been reported too [17]. However, studies on intracellular changes and metastasis mechanisms of neuron cells in AD related to tau aggregation are still insufficient and requires further investigations for better diagnosis and treatment prior to clinical trials. Additionally, as far as the nano-sized therapeutic delivery systems are concerned, endocytosis plays significant role in cargo uptake and is being rigorously studied in recent years. It is also to note that the understanding of metastasis mechanism in tauopathy in intracellular transport systems is exceedingly important, especially in the light of microtubules. Therefore, further studies about the impact of tau aggregation on microtubule destabilization and transport system damage are required.

Here, modern bioimaging techniques are playing crucial role to accomplish these studies where various dye-tagged molecules are being continuously employed as traditional practices [18]. However, to investigate such mechanistic pathways these dye-tagged molecules often need to be irradiated with high energy (visible, UV, etc.) photons and bears the risk of producing lesion in neuron cells under investigation [19]. Situation becomes more challenging when such investigations are time consuming and the neuron cells to be investigated are already known as vulnerable under harsh conditions, e.g., cytotoxicity caused by incident laser power, etc. can easily hinder the study. At this juncture upconversion nanoparticles (UCNPs), which can be excited with low energy near infrared (NIR) radiation, appears to be a promising candidate [20]. UCNPs are lanthanide or actinide doped rare earth element-based nanocrystals and are being increasingly used in the area of in vivo and in vitro bioimaging, biosensing, photodynamic therapy, nanomedicines, etc. They have high optical penetrating capability in tissue. Excitation of these nanoparticles by NIR upconverts the energy of these NIR photons (e.g., 980 nm) and emit visible or UV radiation. These particles show negligible biological background fluorescence and are being increasingly used as effective optical imaging contrast agents. They have longer luminescence lifetime and can be easily synthesized in a range of sizes starting from sub-10 nm dimension [21]. Additionally, ease of their surface functionalization by small or macro molecules via most popularly used ligand exchange method provides the opportunity to prepare aqueous colloidal dispersion which can easily be used in biological media without the concern of cytotoxicity. Surface functionalization of these UCNPs can not only enhance their colloidal stability but also provides the opportunity to load various types of therapeutics, e.g., hydrophilic, hydrophobic, micro-, and macro-molecules. These UCNPs are also being increasingly used to permeate blood-brain barrier and deliver therapeutics at the neuron cell or execute photothermal therapy [22]. These surface functionalized nanoparticles can be foreseen as excellent fluorescent and/or magnetic nanoprobes to revolutionize the non-invasive bioimaging industry.

In this study, tau aggregation was induced in SH-SY5H human neuroblastoma cells by treating the specific activators of PKA, forskolin and PP2A-inhibiting okadaic acid. Approximately, ~100 nm sized hexagonal UCNPs co-doped with rare earth elements erbium and ytterbium were synthesized and their surfaces were functionalized using polyacrylic acid which improved their colloidal stability and introduced negative surface charges. These particles were allowed to internalize by the tau aggregated neuroblastoma cells and their influence on the intracellular transport mechanism, such as cellular uptake, endocytosis and exocytosis processes were explored.

## 2. Materials and Methods

### 2.1. Materials

Erbium (III) chloride (ErCl_3_, 99.9%, anhydrous powder), yttrium (III) chloride (YCl_3_, 99.99%, anhydrous powder), ytterbium (III) chloride (YbCl_3_, 99.99%, anhydrous powder), ammonium fluoride (≥98.0%, ACS reagent), 1-octadecene (ODE, 90%, technical grade), oleic acid (OA, 90%, technical grade) and poly(acrylic acid) (PAA, ~450,000 g/mol), were purchased from Sigma-Aldrich(Goyang, Korea). Sodium oleate (>97%) was obtained from Tokyo Chemical Industry (TCI) (Seoul, Korea). Ethyl alcohol (99.9%, GR grade), cyclohexane (extra pure grade) and *N*,*N*-dimethylformamide (DMF, 99.5%) were purchased from Duksan Reagent while nitrosonium tetrafluoroborate (NOBF_4_, 98%) was obtained from Alfa Aesar (Seoul, Korea). All the materials were used as received without any further purification process. Water used in this work was ultrapure obtained from a Milli-Q filtration system.

### 2.2. Synthesis of UCNPs

#### 2.2.1. Core Synthesis

The lanthanide doped NaYF_4_ core nanoparticles were synthesized via solvothermal synthetic route following the previously reported procedure with some modifications [21]. Lanthanide precursor solution was prepared in a 3-necked round bottom flask having YCl_3_ (0.39 mmol, 76.1 mg), YbCl_3_ (0.1 mmol, 27.9 mg) and ErCl_3_ (0.01 mmol, 2.8 mg) to which OA (2.7 g) and ODE (5.5 g) were added. The reaction mixture was heated for an hour at 110 °C with constant stirring allowing the materials to result clear solution. Air inside the flask was then replaced with dry Ar-gas and the purging continued. Sodium oleate (1.25 mmol, 380.5 mg, purity was taken into consideration during the mass calculation) and NH_4_F (2 mmol, 74.1 mg) were added to it keeping the whole set up under the Ar-gas atmosphere. The flask was heated for 75 min at 310 °C. Once the reaction is complete, the heating mantle was removed cautiously, and the flask was cooled rapidly by the flow of cold air. Once the temperature reached ~75 °C, excess ethanol was added to it to cause precipitation of thus synthesized core nanoparticles composed of NaYF_4_: 20%Yb, 2%Er. This product solution was centrifuged for 5 min at 3400× *g* (Combi 514R, Hanil Science Industrial, Gimpo, Korea) to collect the core nanoparticles. The supernatant was discarded, and the nanoparticle pellets were re-dispersed in cyclohexane via ultra-sonication (Power Sonic 505, Korea Science Corporation, Gyeonggi-do, Korea). Once the particles were well-dispersed, excess of ethanol was added to cause re-precipitation. The solution mixture was centrifuged, and the supernatant was discarded. This process of washing the core nanoparticles were repeated for a total of three times to remove surfactants and any sodium fluoride (NaF) formed during the reaction. Finally, the washed core nanoparticles were dispersed in cyclohexane via sonication and stored at 4 °C for further use.

#### 2.2.2. Epitaxial Growth of Shell on Core

Undoped NaYF_4_ shells were designed on the earlier synthesized core nanoparticles via epitaxial growth following slight modification of the method mentioned elsewhere [23]. Briefly, in a dry 3-necked round bottom flask having YCl_3_ (0.25 mmol, 48.8 mg), oleic acid (6 mL) and 1-octadecene (15 mL) were added and the mixture was heated for an hour at 110 °C under vacuum. Once a clear solution was achieved, the temperature was reduced to 60 °C this is when the earlier prepared core dispersion in hexane (5 mL) was injected and the reaction mixture was stirred at this temperature for another hour or till the hexane got removed. Finally, vacuum was broken by introducing Ar-gas into the flask and the solution was allowed to reach room temperature. After the addition of solid anhydrous NH_4_F (1 mmol, 37 mg) and sodium oleate (0.625 mmol, 190.3 mg), the reaction mixture was again heated for 1 h at 310 °C. The solution was cooled rapidly to reach 75 °C at which an excess of ethanol was added to cause precipitation. This product solution was then transferred to a centrifuge tube and washed thrice following the procedure (centrifugation and re-dispersion) used earlier during core purification. Finally, these core-shell nanoparticles (NaYF_4_: 20% Yb, 2% Er @ NaYF_4_) which were stabilized by the presence of oleate chains at their surfaces (UCNP@OA), re-dispersed in cyclohexane and stored at 4 °C for next use. The concentration of this dispersion was measured gravimetrically.

### 2.3. Surface Functionalization of UCNPs by Polymer

#### 2.3.1. Surface Modification of UCNP@OA

The surfaces of UCNPs were modified via a two-step method to improve their long-term stability in aqueous media and biocompatibility for the uptake by cells [24]. Firstly, the oleate (OA) on UCNP surfaces were substituted by BF_4_^−^. In a glass vial, earlier prepared hexane dispersion of UCNP@OA (2 mL, 15 mg mL^−1^) was added dropwise to a freshly prepared solution of NOBF_4_ in DMF (5 mL, 5 mg mL^−1^) and stirred vigorously for about 10 min at 30 °C. This dispersion was allowed to settle till a phase separation occurred where DMF containing the UCNP@ appeared at the lower phase (pale yellow colored) which was collected and an excess of chloroform (5 times by volume) were added to cause precipitation of these surface modified UCNPs. This dispersion was centrifuged for 5 min at 1000× *g* and the supernatant was discarded. The pellets were re-dispersed in DMF by sonication and then the process of precipitation and centrifugation repeated for two more times to wash away unreacted NOBF_4_ molecules. Finally, these UCNP@BF_4_^−^ particles were re-dispersed in DMF (6 mL) and concentration was determined to be ~5 mg mL^−1^.

In the second step, UCNP@BF_4_^−^ were treated with polymer PAA to prepare UCNP@PAA. In a glass vial having 1 mL of deionized water, 100 mg of PAA was dissolved by constant stirring and the pH was adjusted to 7 by 0.1 N NaOH solution. 1 mL of the UCNP@BF_4_^−^ in DMF, prepared in the earlier step, was added dropwise into the aqueous solution of PAA. pH of this reaction mixture was re-confirmed to be around 7 and stirred for 30 min at room temperature. Finally, this dispersion was centrifuged at 21,000× *g* (CF-10, Daihan Science, Seoul, Korea) for 15 min and the pellets were collected while the supernatant was discarded. These pellets of UCNP@PAA were re-dispersed in aqueous media via sonication. This process of washing (centrifugation and re-dispersion) were repeated thrice to ensure the absence of unbound polymer. The UCNP@PAA dispersion was then filtered through a disposable Cellulose Acetate membrane (DISMIC-13CP, Advantec, Niigata, Japan) of pore diameter 0.45 µm to refute the presence of any aggregates and were stored at 4 °C for further experiments.

#### 2.3.2. Characterization

The morphology and size of synthesized UCNPs were investigated using a multifunctional field-emission transmission electron microscope (TEM, TECNEI G2 F20 [FEI], KBSI Gwangju Center, Gwangju, Korea) with an accelerating voltage of 200 kV. The sample for TEM experiment were prepared by dropping 8 µL of sonicated UCNP dispersion (200 µg mL^−1^ in cyclohexane) on a carbon coated copper grid (200 mesh, Ted Pella, Inc., Redding, CA, USA). A 980 nm CW laser (EM595, Gooch & Housego, Nagoya, Japan) was used to record the emission spectrum of nanoparticle dispersion. FTIR spectra of UCNP@OA and UCNP@PAA samples were recorded on a Nicolet iS10 Spectrometer (Thermo Fisher Scientific, Seoul, Korea) over the range of 4000 to 500 cm^−1^ using the KBr pellet technique and a frequency resolution of 4 cm^−1^. Hydrodynamic size and *ζ*-potential measurements of UCNPs functionalized by PAA were recorded as aqueous solutions using an ELS 8000 (Photal, Otsuka Electronics Co. Ltd., Osaka, Japan).

### 2.4. Cellular Uptake and Exocytosis

#### 2.4.1. Cell Culture

SH-SY5Y human neuroblastoma cells were obtained from Korea Cell Line Bank and cultured in Dulbecco’s Modified Eagle’s Medium (DMEM; Gibco) supplemented with 10% fetal bovine serum (FBS) and 1% of Penicillin streptomycin in 100 mm cell culture dish at 37 °C and 5% CO_2_.

#### 2.4.2. Cell Seeding and UCNP Treatment

For uptake and co-localization experiments, cells (1 × 10^5^ cells mL^−1^, 1 mL suspension) were seeded in confocal dishes (SPL) pre-treated with attaching molecule. Once the cells are attached (~24-h of incubation) to dish surfaces, cells in one dish were treated with 40 µM of forskolin (Sigma,) while cells in other dish with 15 nM of okadaic acid and incubated for 24 h to induce tau aggregation. Simultaneously, the control group was prepared by treating the samples with 2 µL of dimethyl sulfoxide (DMSO; Sigma) for the same duration (24 h). All these three groups of cell samples were treated with 20 µL (40 µg mL^−1^) of UCNPs for 20 min followed by washing with PBS. All these samples were investigated for the incubation durations of 20 min, 2 h, and 24 h, i.e., there were three groups of samples and each group of samples were having three samples with incubation time of 20 min, 2 h, and 24 h, thus a total of 9 samples were investigated.

#### 2.4.3. Cell Staining and Imaging

To stain cell organelles, early endosome and late endosome staining dye (Bacmam 2.0, Thermos fisher scientific), Lysotracker and NucBlue™ Live ReadyProbes™ reagents were used following the instruction noted in protocols provided by manufacturers. All the earlier mentioned nine samples were immobilized following the 4% paraformaldehyde fixation method. Finally, fixed and stained cells were imaged using a Nikon Eclipse Ti2 microscope (Nikon, Korea) coupled with 980 nm CW laser (EM595, Gooch & Housego).

## 3. Results and Discussion

### 3.1. Characterization of Synthesized Nanoparticles

**Figure 1:**
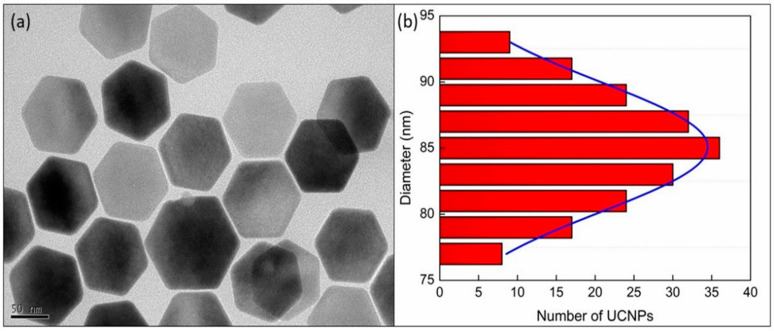
Investigation of size and shape of the core-shell upconversion nanoparticles as obtained by (**a**) transmission electron microscopy image (scale bar 50 nm) and (**b**) the size distribution of the nanoparticles as obtained from the TEM image along with the fit (blue line) by Gaussian distribution equation.

The size and geometry of the core-shell UCNPs (β-NaYF_4_:Yb^3+^, Er^3+^@NaYF_4_) were evaluated by investigating the TEM image as presented in Figure 1 which shows the formation of hexagonal UCNPs with an average dimension of ~85 nm (Figure 1a) (~97 nm as measured by DLS. Size distribution of these core-shell nanoparticles, as obtained from their corresponding TEM images, are shown in Figure 1b. Analysis of the statistical distribution of nanoparticles confirms the size. The fitting of the size distribution graph by Gaussian distribution equation evaluated the average diameter of core-shell UCNPs as 85.08 ± 0.08 nm.

**Figure 2:**
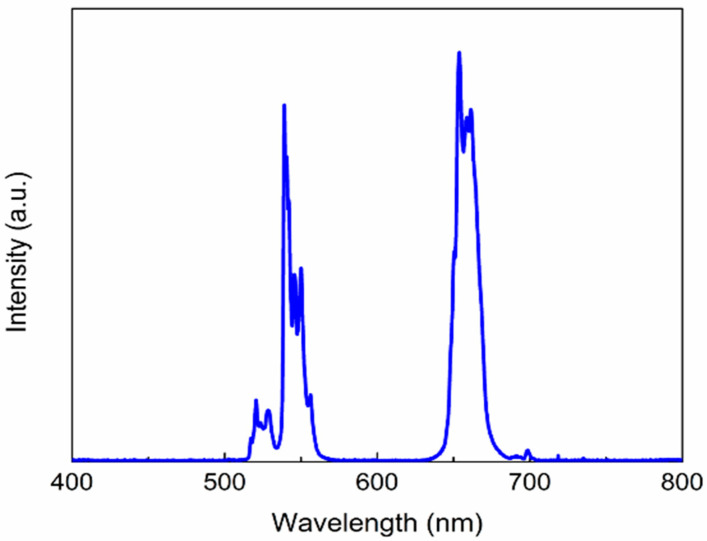
The emission spectrum of core-shell (β-NaYF_4_: 20%Yb^3+^, 2%Er^3+^@NaYF_4_) upconversion nanoparticles showing their peak positions obtained by exciting the nanoparticles with 980 nm CW laser (375 mW cm^−2^, 100 ms).

**Figure 3:**
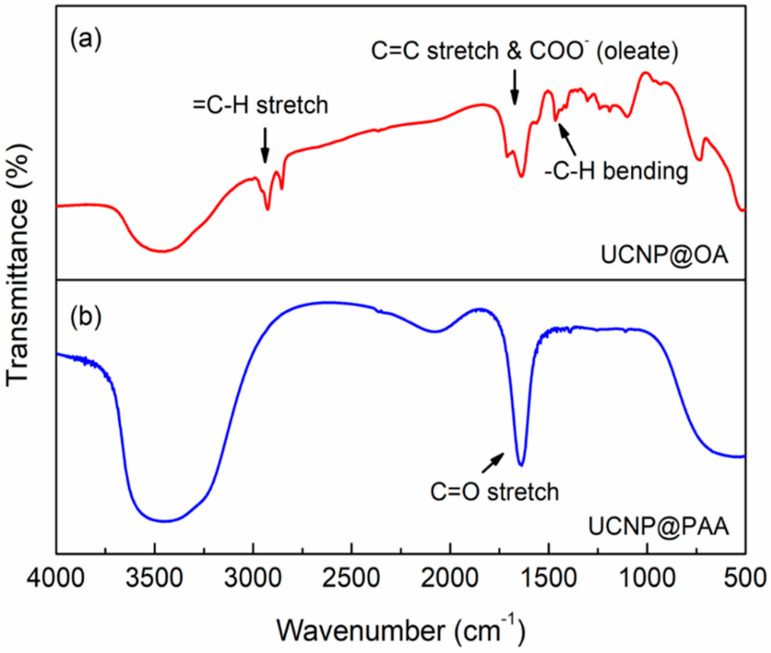
FT-IR spectra of (**a**) UCNPs capped with oleate chains formed during their synthesis and (**b**) UCNP surfaces functionalized by polyacrylic acid (PAA). The spectra were obtained using 16 scans at the resolution of 4 cm^−1^ via the KBr technique.

The emission spectrum of these core-shell UCNPs, as presented in Figure 2, was recorded by exciting the particles using 980 CW laser with a power of 375 mW cm^−2^ and an exposure time of 100 ms. The success of surface modification on UCNP core-shell nanoparticles by PAA was confirmed by Fourier transform infrared spectroscopy. The FTIR spectra of pristine oleate-capped UCNPs and the PAA modified UCNPs are presented in Figure 3. The peaks appeared at 2926, 1637 and 1466 cm^−1^ of the spectrum of UCNP@OA (Figure 3a) represent =C–H stretch, CC stretch & COO–, and –C–H bending of the oleate chains. On the other hand, in spectrum of UCNP@PAA (Figure 3b), the absence of peak at 2926 cm^−1^ (=C–H stretch) and the presence of the strong peak at 1636 cm^−1^ for the C=O stretching of polyacrylic acid whose each repeating unit has one –COOH group, show that the functionalization was achieved successfully. Beside this, the presence of broad peaks at both the spectra are due to the O–H stretching vibration of water molecules that might have got absorbed on samples during the measurements.

**Figure 4:**
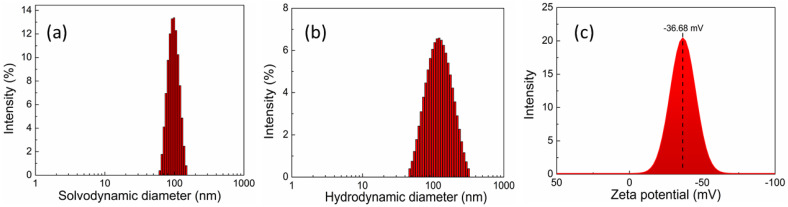
(**a**) The size of oleic-functionalized UCNPs measured in hexane and (**b**) the hydrodynamic size of UCNPs surface functionalized by polyacrylic acid measure in in PBS buffer (1×) at pH 7. (**c**) The zeta potential of UCNPs surfaced functionalized by polyacrylic acid and measured in PBS buffer at pH 7.

The hydrodynamic size of UCNPs functionalized by PAA and their surface charge after the functionalization are investigated by the dynamic light scattering and zeta potential analysis. The measurements were carried out using the particles of concentration approximately 0.05 mg mL^−1^ in PBS buffer (1×) at pH 7.4. The hydrodynamic size of the PAA functionalized UCNPs was found to be 112 ± 1.2 nm (Figure 4a) and the surface charge was −36.6 ± 1.2 mV (Figure 4b) which has also contributed to its colloidal stability.

### 3.2. Cellular Uptake and Exocytosis in Tau-Aggregated Cell

SH-SY5Y human neuroblastoma cells were treated with forskolin and okadaic acid to induce two types of tau aggregations. As reported elsewhere, treatment of these cells with 20 µM of forskolin for 24 h can induce sufficient aggregation of tau proteins [25]. It is known that the aggregation induced by 30 nM of okadaic acid is more efficient when compared to that of the forskolin for 24 h. Hence, in order to investigate and compare impacts caused by both of these inducers, the concentration of forskolin was used as 40 µM while the concentration of okadaic acid was reduced to 15 nM. To confirm the uptake and internalization of big sized UCNPs, early endosome, late endosome and lysosome were stained. These stained cells were divided into three groups: (1) cells treated with UCNPs for 20 min and (2) the cells treated with UCNPs for 20 min followed by washing and incubating for 2 h and (3) 24 h. Finally, to evaluate the internalization of UCNPs, 3D images of cells were investigated.

**Figure 5:**
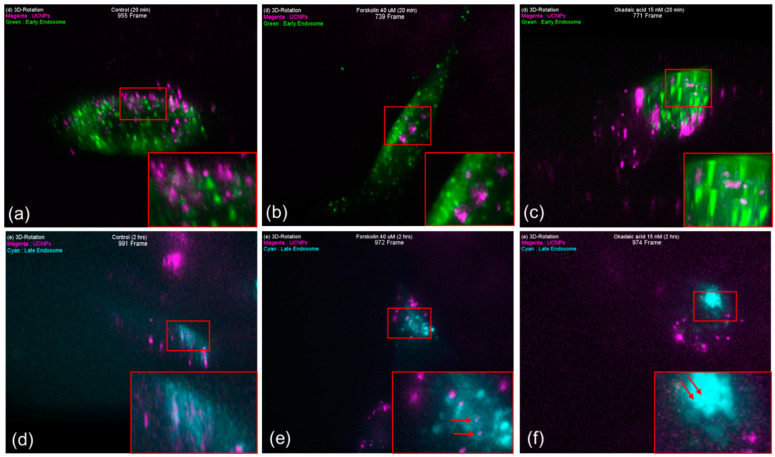
Early state colocalizations of UCNPs with early endosomes or late endosomes in tau aggregated SH-SY5Y cell. (**a**) Control 20 min (955 frame), (**b**) forskolin 20 min (739 frame), (**c**) okadaic acid 20 min (771 frame), (**d**) control 2 h (991 frame), (**e**) forskolin 2 h (972 frame), and (**f**) okadaic acid 2 h (974 frame). Magenta: UCNPs, green: early endosome, cyan: late endosome.

It was found that the induced tau aggregated SH-SY5Y cells, which were treated with UCNPs for 20 min, have shown good uptaking capability for all three cases, namely, cells treated with (a) DMSO (as control), (b) forskolin, and (c) okadaic acid. Through 2D images (Figure S1), it is apparent that a large amount of uptaken UCNPs are co-localized with early endosome, late endosome, and lysosome; however, investigation of the reconstructed 3D images confirmed that most of the uptaken UCNPs were not co-localized (Figure 5a–f and Videos S1–S3).

Generally, the endocytic pathway refers to the track through which an internalized molecule can travel from the plasma membrane to the lysosome, and 10–200 nm sized UCNPs can enter the endocytic pathway predominantly mediated by the protein clathrin. Furthermore, since actin filament is involved in uptake process through the plasma membrane, hence it is understood that the aggregation of tau protein could not affect the uptake of UCNPs significantly. Additionally, it has been reported that the endocytic pathway from the plasma membrane to the lysosome can get completed in about 30 min. However, in our studies, it could be seen that longer time was required from internalization to early endosome. The reason for this delay could be that once an UCNP is attached to the cell membrane, the cell performs the work of recognition prior to its internalization. Thereafter, the results obtained from the cells which were incubated for 2 h after the UCNP treatment (Figure S2), showed that the particles were either near the late endosome and lysosome or co-localized (Figure 5d–f and Videos S4–S6). This shows that UCNPs uptaken by the SH-SY5Y cells followed the endocytic pathway.

Since tau protein binds to microtubule and contributes to its stability, when a large amount of tau protein is aggregated, it is anticipated that a problem will occur in the vesicle transport system composed of microtubules and motor proteins. Furthermore, it has been reported that most of UCNPs undergo exocytosis within 24 h of post-treatment via intracellular transport. In order to study the effect of tau aggregation, the SH-SY5Y cells having induced tau-aggregation, were treated with big sized UCNPs for 24 h and the results were analyzed.

**Figure 6:**
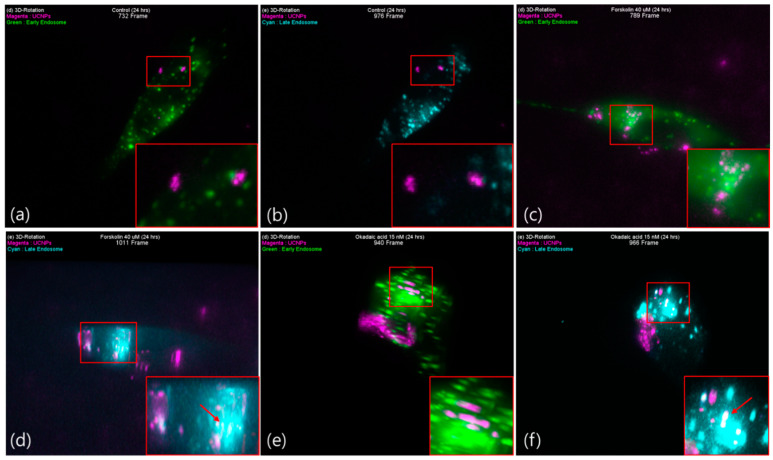
Late state colocalization of UCNPs with early endosomes or late endosomes in tau aggregated SH-SY5Y cell. (**a**) Control 24 h (732 frame), (**b**) Control 24 h (976 frame), (**c**) forskolin 24 h (789 frame), (**d**) forskolin 24 h (1011 frame), (**e**) okadaic acid 24 h (940 frame), and (**f**) okadaic acid 24 h (966 frame). Magenta: UCNPs, green: early endosome, cyan: late endosome.

In case of the control group, it was observed that after 24 h post-treatment, most of the UCNPs undergone exocytosis (Figure 6a,b and Figure S3). Thus, it is clear that our relatively big-sized UCNPs used the normal endocytosis and exocytosis pathways facilitated by vesicles.

**Figure 7:**
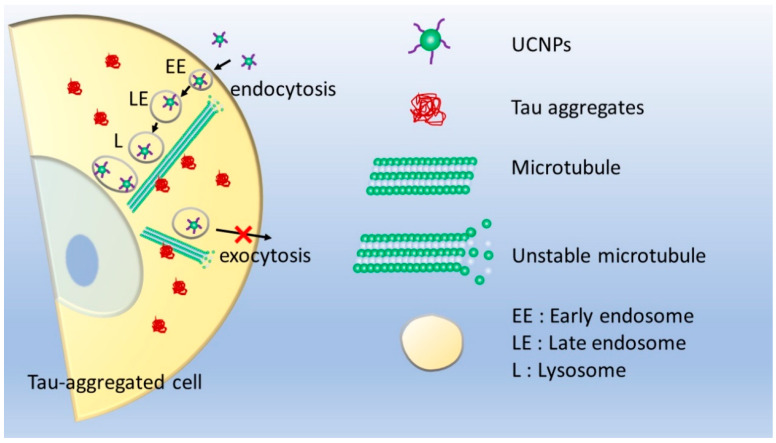
Schematic presentation of endocytosis and exocytosis of ~100 nm size surface functionalized UCNPs in tau-aggregated neuron cells.

In case of cells treated with forskolin (40 µM) and okadaic acid (15 nM), majority of UCNPs remained inside the cells co-localized with late endosome and Lysosome (Figure 6c–f and Videos S7–S9) compared to that of the control. It is known that microtubule, the main road for intracellular transport, does not get completely degraded even if it becomes unstable due to tau aggregation. Thus, even though the transport function is maintained, it is found that exocytosis did not occur as it was in the case of control (Figure 7). This means that further attention is required during the investigation of tau aggregated cell.

Various pH responsive delivery systems are being continuously developed by researchers for the treatment of neuron cells affected by Alzheimer’s disease. During such development, researchers should also consider the fact that such carriers of sizes comparable to the size of UCNPs used in this work, will have the tendency to stay exposed in low pH environment for longer duration demanding the requirement of designing a strategy which would be able to continue to protect the loaded therapeutics at this stage. Furthermore, in the case when a biodegradable carrier is not used, once the delivery is complete it must go out of the cell via exocytosis even if it is not cytotoxic. Therefore, in order to use a suitable delivery system for tau aggregated neuron cells, strategies should be designed considering release duration, release trigger, exocytosis, or degradation of carrier materials, etc.

This study on forskolin and okadaic acid induced tau aggregation can also provide an important information for the metastasis of tauopathy in tau aggregation related Alzheimer’s disease. Although tau protein is an intracellular secreted protein, aggregated tau proteins and tau oligomers are often found freely or wrapped in vesicles around tau aggregated neuron cells. Even though the abnormally aggregated tau protein metastasis mechanisms are being continuously studied, but the major metastasis mechanisms are still unclear. This study confirms that UCNPs of size ~100 nm were well uptaken by both the tau aggregated as well as healthy neuron cells (Figure 5). However, exocytosis of these UCNPs were less effective and remains mostly in the late endosome and lysosome (Figure 6). Recently, Takeda et al., [26] reported that the most effective endocytosed form of tau is spherical. Nobuhara et al., [27] have also reported that tau oligomers are mainly accumulated in the lysosome. Even though the cell lines used in their studies are different than the ones used in this work, both of their reports support the observation found here. This shows that the experimental method and results presented in this work can be useful for the study of metastasis mechanism in tauopathy. Thus, the ease of UCNP synthesis in variety of sizes and their surface modification (e.g., tau aggregated proteins) can present a promising strategy to investigate the metastasis mechanism.

## 4. Conclusions

There are various unexplored challenges in studying tauopathy and their metastasis which requires further attention in cellular-level researches. During the designing of an efficient drug delivery system, the prospect of carrier-induced toxicity and their exocytosis must be considered. Unlike the treatment of cancer cells, where such cytotoxicity is taken advantage of, treatment of tau aggregated neuron cells by drug loaded carriers require detailed attention towards these two points (cytotoxicity and exocytosis) because such carriers are to deliver their cargo to the live neuron cells and recover those. Thus, exploring the carrier-induced toxicity and their exocytosis after delivery are shown to be two very important parameters during the evaluation of a carrier. In this study, polyelectrolyte functionalized UCNPs of ~100 nm were synthesized and used as model carrier for the neuron cells having aggregated tau proteins. To mimic neurodegenerative disease infected neuron cells, firstly tau-aggregation was induced into normal neuron cells using forskolin and okadaic acid. Taking the advantage of UCNPs as bright fluorescent nanoprobes their bioimaging was carried out, which confirmed that even though there was no significant difference in the uptake of such ~100 nm sized nanocarriers in tau aggregated cells, the intracellular transport and exocytosis processes were remarkably affected when compared to that of the normal neuron cells. This strategy can provide interesting information on intracellular transport pathways and tauopathy transfer mechanisms in cellular-level studies of tau-related neurodegenerative diseases. This study also paves the way to design efficient carriers that would not only deliver their cargos effectively but also take part in biodegradation and/or exocytosis, which are often overlooked in many studies.

## Data Availability

All data are available upon request.

## Supplementary Materials

The following are available online at https://www.mdpi.com/1999-4923/13/1/102/s1, Figure S1: 2D colocalization images of UCNPs with early endosomes, late endosomes and lysosomes 20 min after UCNP treatment in tau aggregated SH-SY5Y cell. (Scale bar = 20 µm), Figure S2: 2D colocalization images of UCNPs with early endosomes, late endosomes and lysosomes 2 h after UCNP treatment in tau aggregated SH-SY5Y cell. (Scale bar = 20 µm), Figure S3: 2D colocalization images of UCNPs with early endosomes, late endosomes and lysosomes 24 h after UCNP treatment in tau aggregated SH-SY5Y cell. (Scale bar = 20 µm), Video S1: 3D colocalization images of control group (20 min), Video S2: 3D colocalization images of forskolin treated group (20 min), Video S3: 3D colocalization images of okadaic acid treated group (20 min), Video S4: 3D colocalization images of control group (2 h), Video S5: 3D colocalization images of forskolin treated group (2 h), Video S6: 3D colocalization images of okadaic acid treated group (2 h), Video S7: 3D colocalization images of control group (24 h), Video S8: 3D colocalization images of forskolin treated group (24 h), Video S9: 3D colocalization images of okadaic acid treated group (24 h).
