# Supplementary material for: Uptake of Polyelectrolyte Functionalized Upconversion Nanoparticles by Tau-Aggregated Neuron Cells
Source: Pharmaceutics. 2021 Jan 14;13(1):102. doi: 10.3390/pharmaceutics13010102 (PMC7829809; doi:10.3390/pharmaceutics13010102)
Supplement: Supplementary file 1 [file pharmaceutics-13-00102-s001.zip › pharmaceutics-1076569-suppl/pharmaceutics-1076569-Supplimentary Materials.doc]

Supplementary Materials: Uptake of Polyelectrolyte Functionalized Upconversion Nanoparticles by Tau-Aggregated Neuron Cells

Yo Han Song, Ranjit De and Kang Taek Lee


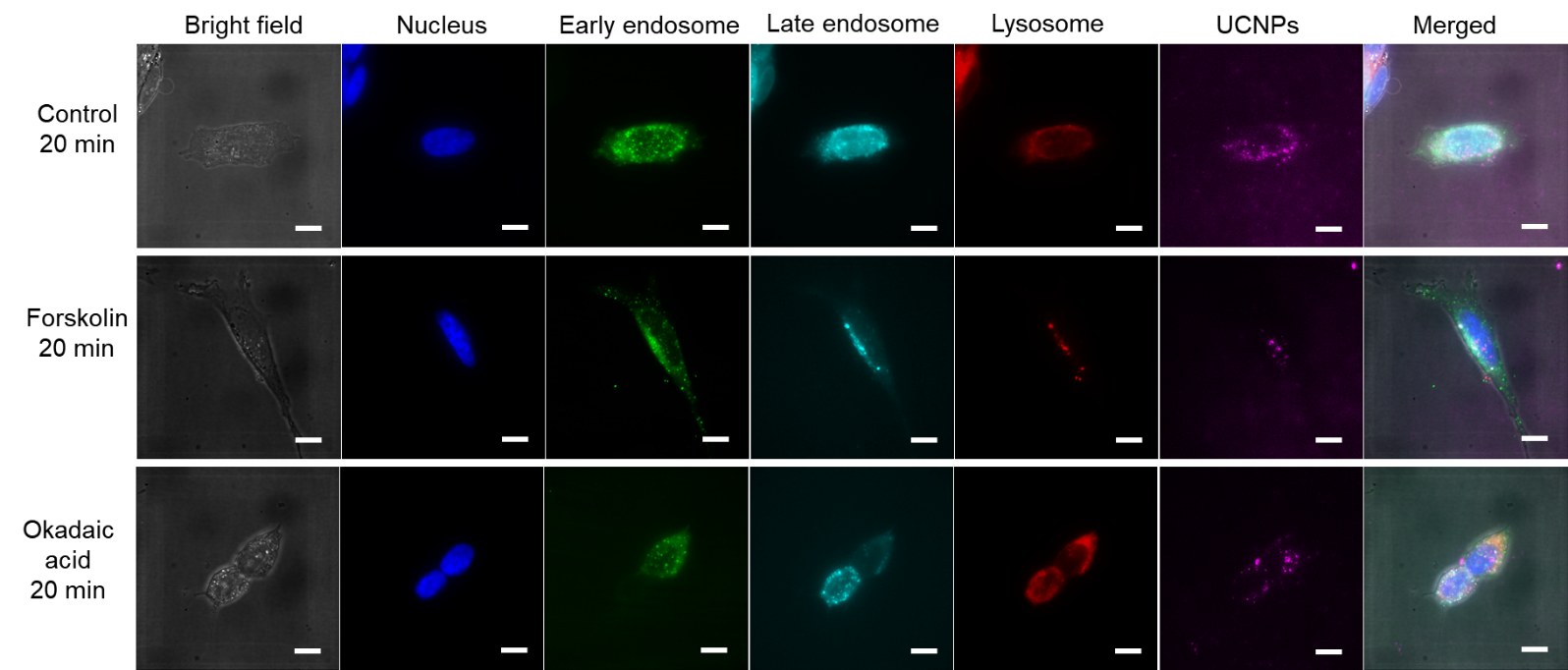


**Figure S1.** 2D colocalization images of UCNPs with early endosomes, late endosomes and lysosomes 20 minutes after UCNP treatment in tau aggregated SH-SY5Y cell. (Scale bar = 20 *µ*m)


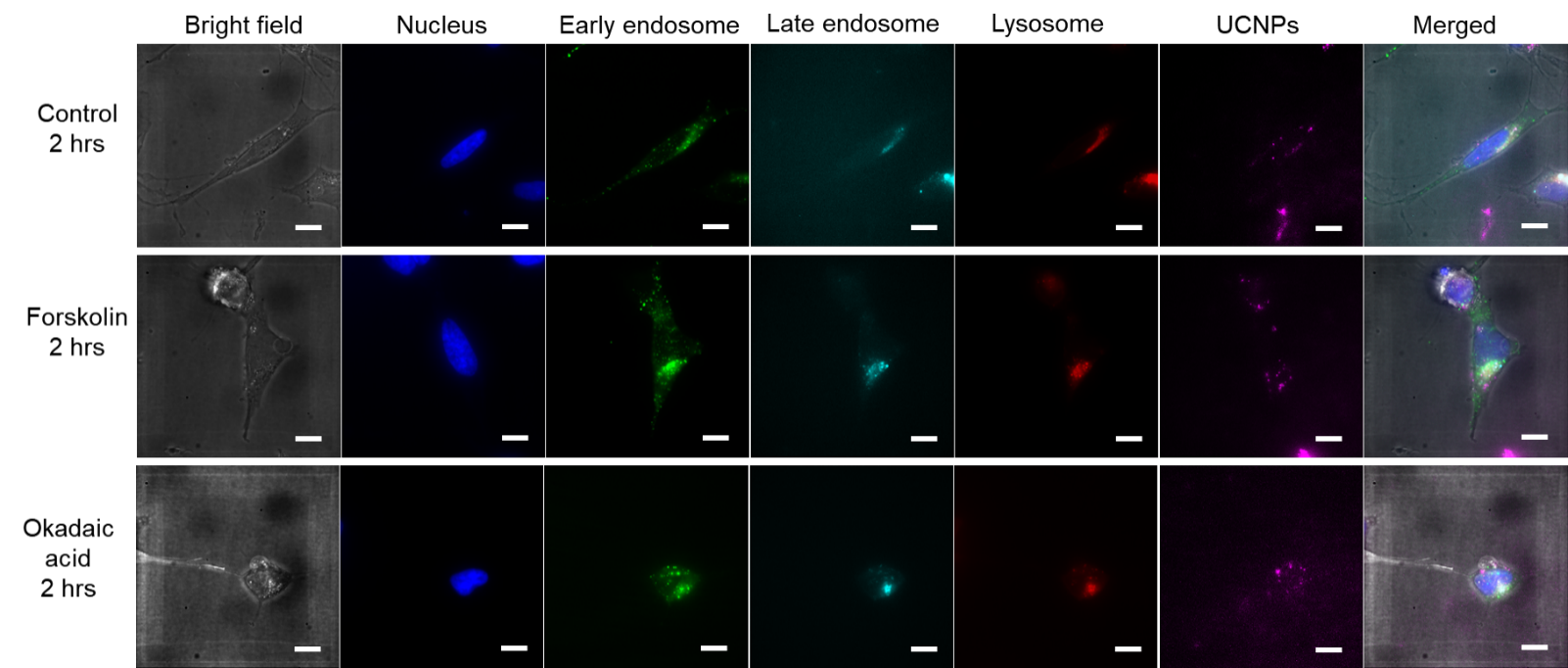


**Figure S2.** 2D colocalization images of UCNPs with early endosomes, late endosomes and lysosomes 2 hours after UCNP treatment in tau aggregated SH-SY5Y cell. (Scale bar = 20 *µ*m)


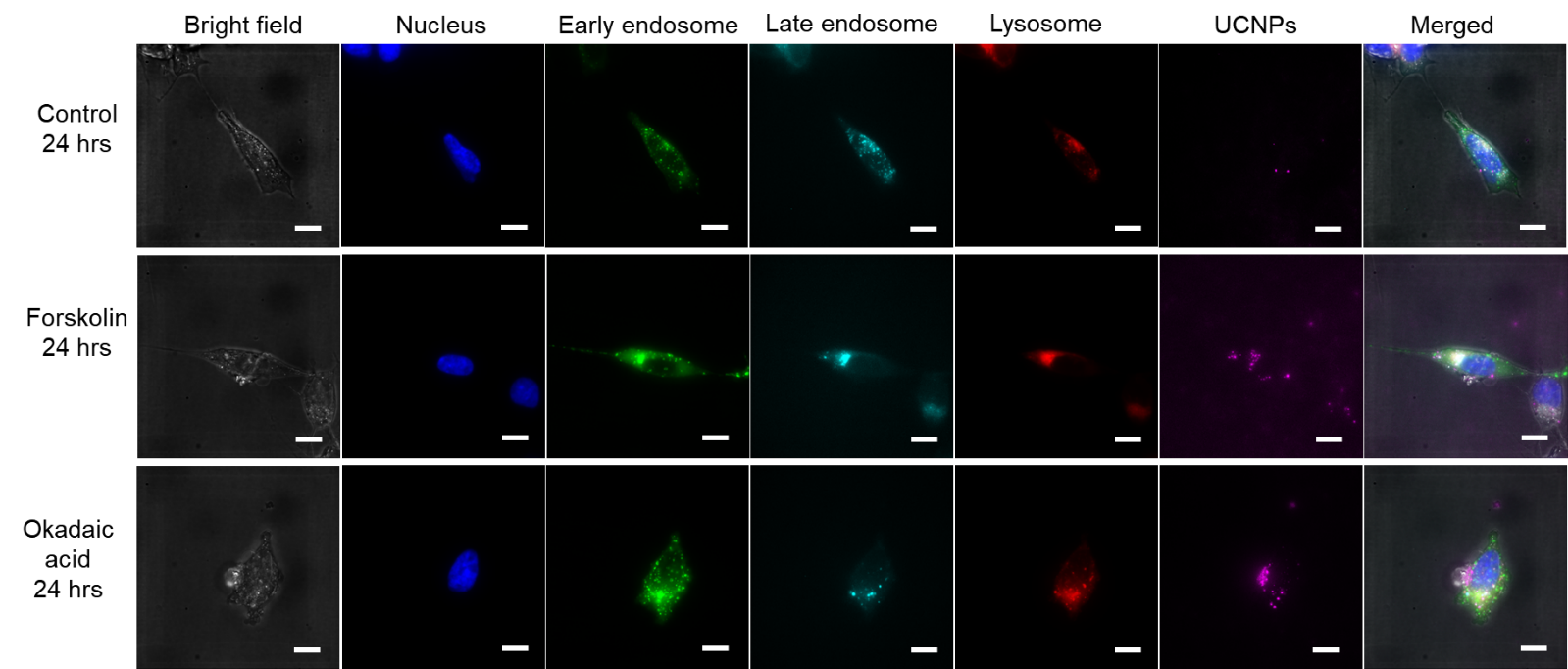


**Figure S3.** 2D colocalization images of UCNPs with early endosomes, late endosomes and lysosomes 24 hours after UCNP treatment in tau aggregated SH-SY5Y cell. (Scale bar = 20 *µ*m)
